# *CYB5D2* inhibits the malignant progression of hepatocellular carcinoma by inhibiting *TGF-β* expression and epithelial-mesenchymal transition

**DOI:** 10.32604/or.2024.050125

**Published:** 2025-02-28

**Authors:** DONG JIANG, ZHI QI, ZHIYING XU, YIRAN LI

**Affiliations:** 1 Department of Ultrasound, Eastern Hepatobiliary Surgery Hospital, The Third Affiliated Hospital of Naval Medical University, Shanghai, 200433, China; 2 Department of Hepatic Surgery IV, Shanghai Eastern Hepatobiliary Surgery Hospital, Third Affiliated Hospital of Naval Medical University, Shanghai, 200433, China; 3 Department of Neurology, Eastern Hepatobiliary Surgery Hospital, The Third Affiliated Hospital of Naval Medical University, Shanghai, 200433, China

**Keywords:** Cytochrome b5 domain containing 2 **(***CYB5D2*), Malignant progression, Hepatocellular carcinoma (HCC), Transforming growth factor beta (*TGF-β*), Epithelial-mesenchymal transition (EMT)

## Abstract

**Background:**

Hepatocellular carcinoma (HCC) is a prevalent liver malignancy. This study examined the roles of transforming growth factor beta (*TGF-β*) and cytochrome b5 domain containing 2 (*CYB5D2*) in HCC etiology and their prognostic biomarker potential.

**Methods:**

Key modules and prognostic genes were identified by analyzing the GSE101685 dataset by weighted gene co-expression network analysis (WGCNA) and Least absolute shrinkage and selection operator (LASSO) Cox regression. The expression levels of *CYB5D2* and *TGF-β* in HCC cell lines were quantified using Quantitative reverse transcription polymerase chain reaction (qRT-PCR) and Western blotting (WB) assays. Effects of *CYB5D2* overexpression on cell proliferation, migration, invasion, and epithelial-mesenchymal transition (EMT) marker regulation were assessed *in vitro*, while *in vivo* tumorigenicity was evaluated using a xenograft model of HCC in nude mice.

**Results:**

In this study, WGCNA identified the turquoise module as significantly associated with HCC, containing 452 DEGs. LASSO Cox regression analysis revealed 9 key prognostic genes, with *CYB5D2* being underexpressed in HCC cells and tissues. *TGF-β* was negatively correlated with *CYB5D2* expression, resulting in poor patient prognosis. Functional assays demonstrated that *CYB5D2* overexpression inhibited proliferation, migration, and invasion of HCC cell lines, and altered EMT marker expression. Furthermore, the addition of *TGF-β* partially reversed the suppressive effects caused by *CYB5D2* overexpression. *In vivo*, CYB5D2 overexpression significantly reduced tumor growth, indicating its potential as a therapeutic target for HCC.

**Conclusion:**

The tumor suppressor function of *CYB5D2* in HCC and its interaction with *TGF-β* offered fresh information on the molecular pathophysiology of HCC and possible treatment avenues.

## Introduction

Approximately 5% of cancer patients are diagnosed with liver cancer, making it the sixth most prevalent cancer type worldwide [1,2]. Annually, there are over 700,000 new cases and approximately 745,000 deaths attributed to liver cancer, positioning it as the second leading cause of cancer-related mortality globally [3]. Hepatocellular carcinoma (HCC) represents the most common form of liver cancer. Research identifies a multitude of primary risk factors contributing to the onset of HCC, including hepatitis B and C virus infections, excessive alcohol consumption, exposure to aflatoxin B1, liver fibrosis, and cirrhosis [4,5]. Although chemotherapy has greatly improved the treatment of HCC, the overall survival (OS) rate is still low and the prognosis is poor [6,7]. Consequently, the development of novel therapeutic approaches and the establishment of reliable early detection techniques are imperative for the clinical management of patients with HCC.

Cytochrome b5 domain-containing 2 (CYB5D2) is a protein that binds heme and promotes neuronal differentiation. It is associated with nervous system development and acts upstream or within the positive regulation of neuronal differentiation [8]. Researchers with relevant experience have verified that *CYB5D2* regulates several malignancies. For instance, Ojo et al. demonstrated that overexpression of *CYB5D2* induces apoptosis in breast cancer cells. Its down-regulation leads to decreased overall survival in breast cancer patients via cellular experiments and survival analysis [9]. Additionally, another study has shown that *CYB5D2* contributes to the prevention of cervical cancer, and upregulation of this gene can prevent the malignant progression of human cervical cancer HeLa cells [10]. Li et al. demonstrated that individuals with cervical cancer who express high levels of *CYB5D2* mRNA have a better prognosis for their condition. This gene mainly reduces epithelial-mesenchymal transition (EMT) by increasing the production of *E-cadherin* [11]. However, the mechanistic role of *CYB5D2* in HCC is not fully understood, necessitating further investigation to elucidate its potential function and impact on HCC progression.

Members of the transforming growth factor-beta (*TGF-β*) family, including *TGF-β1*, *TGF-β2*, and *TGF-β3*, have been documented to act as tumor suppressors. Their functions encompass diminishing cell proliferation, inducing apoptosis, and modulating epithelial-mesenchymal transition (EMT), thereby exerting a profound influence on tumor growth and metastasis through the regulation of various tumor cell behaviors [12,13]. A study indicated that the *TGF-β*/SMAD signaling pathway is linked to a negative prognosis for HCC [14]. Such as C-X-C motif chemokine ligand 6 (*CXCL6*) and *TGF-β* secreted by HCC cells were able to activate the extracellular signal-regulated kinase (ERK) 1/2 signaling pathway in cancer-associated fibroblasts (CAFs), which promoted the release of more CLCF1. This interaction formed a positive feedback loop that accelerated the progression of HCC [15]. *TGF-β* has been studied as a possible therapeutic approach due to its dual involvement in cancer. The purpose of this study was to investigate the involvement of *TGF-β* and *CYB5D2* in tumor growth and HCC. By examining the function of *CYB5D2* and the molecular mechanism of its interaction with *TGF-β*, it may be possible to develop targeted therapies for HCC.

## Materials and Methods

### Animal experiments and human samples

This study was conducted in strict accordance with the recommendations in the Guide for the Care and Use of Laboratory Animals of the Guide for the Care and Use of Laboratory Animals of the National Institutes of Health. All animal experiments were approved by the Ethics Committee of the Third Affiliated Hospital of Naval Medical University (approval number: EHBHKY2014-03-006). Regarding human samples, the study was approved by the Institutional Review Board of Ethics Committee of the Third Affiliated Hospital of Naval Medical University (approval number: EHBHKY2023-KO39-P001). All procedures performed in studies involving human participants followed the ethical standards of the institutional and/or national research committee and with the 1964 Helsinki declaration and its later amendments or comparable ethical standards. Informed consent was obtained from all individual participants included in the study. All samples were anonymized to respect the privacy of participants.

### Microarray data collection and differentially expressed genes (DEGs) analysis

The Gene Expression Omnibus (GEO; https://www.ncbi.nlm.nih.gov/geo/, accessed on 23 August 2023) provided the GSE101685 microarray dataset, which comprises eight groups of normal liver tissues and 24 groups of HCC tissues. The probes were then summarized using Affymetrix annotation files using the “Affy” R package’s median polished probe set. DEGs were identified using the GEO2R tool, with a fold change (FC) >2 or <0.5 and *p* < 0.05 used to identify up-regulated DEGs and down-regulated DEGs.

### Gene co-expression network in the GSE101685 dataset

The co-expression network of DEGs in the GSE101685 dataset was created using the Weighted gene co-expression network analysis (WGCNA) program in R. We first calculated the paired gene Pearson correlation matrix and then used the power function am = |cmn| to get the weighted adjacency matrix. Using the soft threshold power β, we identified weak and strong connections between genes. We also generated a topological overlap matrix (TOM) using the adjacency matrix. Genes with similar expression patterns were assigned to modules using ALM-based average linkage hierarchical clustering. The key module was chosen based on the strongest association between GSE101685 samples and gene modules.

### Batch survival analysis

RNAseq data was obtained from 371 individuals diagnosed with liver hepatocellular carcinoma (LIHC) through The Cancer Genome Atlas (TCGA; https://tcga-data.nci.nih.gov/tcga, accessed on 25 August 2023) database. Subsequently, the “forestplot” program was utilized to conduct a batch survival analysis for the genes in the key module, and the effect of each gene on disease-free survival (DFS) was visualized. Statistical significance was determined using *p*-values less than 0.05. The log-rank test and univariate Cox regression were used to calculate the hazards ratios (HR) and their 95% confidence intervals (CI).

### Construction of signature prognosis model of the turquoise module

The “glmnet” package in R software was utilized to investigate 56 genes in the turquoise module. The least absolute shrinkage and selection operator (LASSO) model was tuned using ten-fold cross-validation to determine the tuning parameters. The genes with the minimum criterion of tunning parameter (*λ*) were identified as the most predictive. The selected genes represent the most statistically significant predictors in the GSE101685 dataset and form the basis of the prognostic model. The LIHC cohort from the TCGA database was then separated into two categories according to the expression pattern of significant genes: high-risk and low-risk, respectively. Both cohorts underwent a risk assessment, and the DFS probability was calculated using a Kaplan-Meier (KM) analysis for each data set. In addition, the median survival time was calculated, and a log-rank test was employed to examine the statistical significance of the survival differences between the two cohorts, yielding a *p*-value. To provide additional context for the comparative risk, the HR for the high-risk group was also calculated. Lastly, the predictive ability of the risk model for patient 1-, 3-, and 5-year survival was assessed using the “timeROC” program, and the Area Under Curve (AUC) analysis was used to create the receiver operating characteristic (ROC) curve. It is worth noting that a higher AUC value suggests better prognosis-predicting ability.

### Construction of a prognostic nomogram and survival analysis

After selecting nine genes with prognostic significance from our risk model, both univariate and multivariate Cox regression analyses were applied to these genes and clinical factors, including age, pT stage, pTNM stage, and tumor grade. Utilizing the “forestplot” package within R, these analyses facilitated the graphical representation of *p*-values, hazard ratios (HRs), and their 95% confidence intervals (CIs). For variables demonstrating significance (*p* < 0.05), a nomogram was developed to assess their influence on patient survival outcomes.

### Differential expression and prognostic significance of CYB5D2 and TGF-β in LIHC

*TGF-β* is known to act as a tumor suppressor in the early stages of HCC [16] by inhibiting cell proliferation and promoting apoptosis, thus preventing the initiation and progression of this malignancy [17]. In this study, we evaluated *CYB5D2* expression levels in both normal liver and TCGA-LIHC samples through the Wilcoxon test, followed by an analysis of the association between *CYB5D2* and *TGF-β*. Additionally, *TGF-β* expression in TCGA-LIHC samples was assessed using the Wilcoxon test to explore its role in LIHC pathogenesis. Finally, the impact of *TGF-β* expression variations on patient OS was determined via KM survival analysis.

### Immunohistochemical analysis of CYB5D2 and TGF-β in the human protein atlas (HPA) database

The HPA (https://www.proteinatlas.org/, accessed on 5 September 2023) database provides a vast collection of immunohistochemical images of human proteins in different tissues and cells, allowing for the exploration of protein distribution and expression patterns. In this database, immunohistochemical analysis was performed to assess the protein expression levels of CYB5D2 and *TGF-β* in both normal liver and HCC tissues.

### Cell culture and transfection

Shanghai Institute of Cell Biology (Shanghai, China) provided the human HCC cell lines C3A (obtained from ATCC), HepG2, MHCC97H, and Hep3B, as well as the human normal liver cell line QSG7701. These cells were grown in RPMI 1640 media with 10% fetal bovine serum (FBS) added, and they were kept at 37°C in a 5% CO_2_ humidified environment. The CYB5D2 overexpression plasmid was transfected into C3A and HepG2 cell lines for the transfection process, while the empty vector plasmid served as the control. Additionally, to investigate relevant cellular responses, 5 ng/mL of the active form of *TGF-β* was added to the medium of C3A and HepG2 cell lines. The transfection process utilized Lipofectamine 3000 (Invitrogen, Carlsbad, CA, USA), adhering strictly to the provided guidelines.

### Quantitative reverse transcription polymerase chain reaction (qRT-PCR) assay

Total RNA was isolated from cells using TRIzol reagent (Invitrogen), following the accompanying protocol. RNA concentration and purity were assessed using a NanoDrop 2000 spectrophotometer. cDNA synthesis was conducted using the PrimeScript RT Reagent Kit (Takara Bio, Otsu, Japan), as per the manufacturer’s directions. The same kit was also used for reverse transcription. qRT-PCR was carried out using SYBR Green PCR Master Mix (Takara) and specific primers for *CYB5D2*, *TGF-β*, *E-cadherin*, *N-cadherin*, *Snail*, and *Twist*, detailed in Table S1. *GAPDH* served as the internal control, and gene expression levels were determined by the 2^−ΔΔCt^ method.

### Western blotting (WB) assay

Protein lysates from cells were prepared using RIPA lysis buffer (Beyotime, Shanghai, China) supplemented with a protease inhibitor cocktail and a phosphatase inhibitor cocktail (both from Roche). Protein concentrations were quantified using the BCA protein assay kit from Thermo Fisher Scientific. Equal amounts of proteins were then subjected to separation via 10% SDS-PAGE gels, followed by their transfer onto PVDF membranes. After blocking for an hour with 5% nonfat milk in TBST, the membranes were incubated overnight at 4°C to be incubated with primary antibodies *CYB5D2* (Invitrogen, #PA5-69862), *TGF-β* (Invitrogen, #MA5-15065), E-cadherin (Invitrogen, #13-1700), N-cadherin (Invitrogen, #33-3900), Snail (Invitrogen, #MA5-14801), and Twist (Invitrogen, #PA5-49688), they were diluted at a 1:1000 dilution. Horseradish peroxidase-conjugated secondary antibodies were then incubated on TBST-washed membranes for one hour at room temperature. Protein bands were visualized and quantified using the Millipore ECL Plus kit and Image J software.

### Immunohistochemistry (IHC)

To evaluate the expression level of CYB5D2, IHC analysis was performed on tissue samples obtained from the Third Affiliated Hospital of Naval Medical University, including HCC and surrounding normal liver tissue. The sections were rehydrated in a graded ethanol series after being deparaffinized in xylene. Antigens were extracted from citrate buffer (pH 6.0) using microwave heating. 3% hydrogen peroxide and 5% bovine serum albumin were used to block nonspecific binding and endogenous peroxidase activity, respectively. Sections were treated with a 1:1000 solution of a primary antibody specific to CYB5D2 for an entire night at 4°C. Hematoxylin staining was used to visualize the nuclei. Finally, stained slices were imaged to evaluate CYB5D2 expression between tumor and normal tissue.

### Cell counting kit (CCK-8) assay

Cellular proliferation was assessed using the CCK-8 test (Dojindo). Transfected cells were cultured in 96-well plates for 24, 48, 72, and 96 h each. After that, each well received a 10 μL aliquot of CCK-8 solution and incubated at 37°C. The absorbance was then measured at 450 nm using a microplate reader.

### Flow cytometry

For the cell cycle assessment, cells were collected, rinsed with chilled phosphate-buffered saline (PBS), and preserved in 70% ethanol at −20°C overnight. Post-fixation, cells were washed with PBS and incubated with propidium iodide (Sigma-Aldrich) in darkness for 30 min to stain DNA. Subsequently, DNA content in stained cells was quantified using a BD Biosciences flow cytometer. Data acquired from the cytometer was processed and analyzed using FlowJo software to determine cell cycle distribution.

### Transwell assays

In transwell chambers with or without Matrigel (Corning Inc., Corning, NY, USA), cell migration and invasion were assessed. The trypsinized cells were resuspended in a medium devoid of serum after being cleaned. 3 × 10^4^ cells were injected into the upper chamber of each Transwell. The lower chamber was filled with 200 μL of serum-free media and 600 μL of chemoattractant-containing medium with 10% FBS. Following a 24-h incubation period, cells passing through the bottom wells of the membrane were fixed with 4% paraformaldehyde and stained with crystal violet. Five randomly chosen areas inside each well were counted for the quantity of migrating and invasive cells under the microscope.

### Xenotransplantation experiments

The Ethics Committee of the Third Affiliated Hospital of Naval Medical University approved all animal treatments (approval number: EHBHKY2014-03-006). Six-week-old male BALB/c nude mice were obtained from SLAC Laboratory in Shanghai, China. In their lateral axillary region of the forelimb near the back, the mice received subcutaneous injections of either empty control C3A cells or C3A cells that overexpressed *CYB5D2*. After 2–3 weeks, the size and weight of the mouse tumors were measured and compared before and after injection to evaluate the impact of *CYB5D2* overexpression on tumor development. Following the final measurement, the mice were euthanized 7 days after the last treatment using CO₂ inhalation at a flow rate of 20% chamber volume per minute until loss of consciousness, followed by cervical dislocation. All euthanasia procedures adhered to the guidelines of the Ethics Committee of the Third Affiliated Hospital of Naval Medical University and followed the American Veterinary Medical Association (AVMA) Guidelines for the Euthanasia of Animals.

### Statistical analysis

Data processing was conducted utilizing R language packages, employing either Student’s *t*-test or one-way Analysis of Variance (ANOVA) for statistical analysis. A threshold of *p* < 0.05 was set for significance determination. The data was provided as mean ± SEM.

## Results

### Identification of a key module in the gene co-expression networks

**Figure 1:**
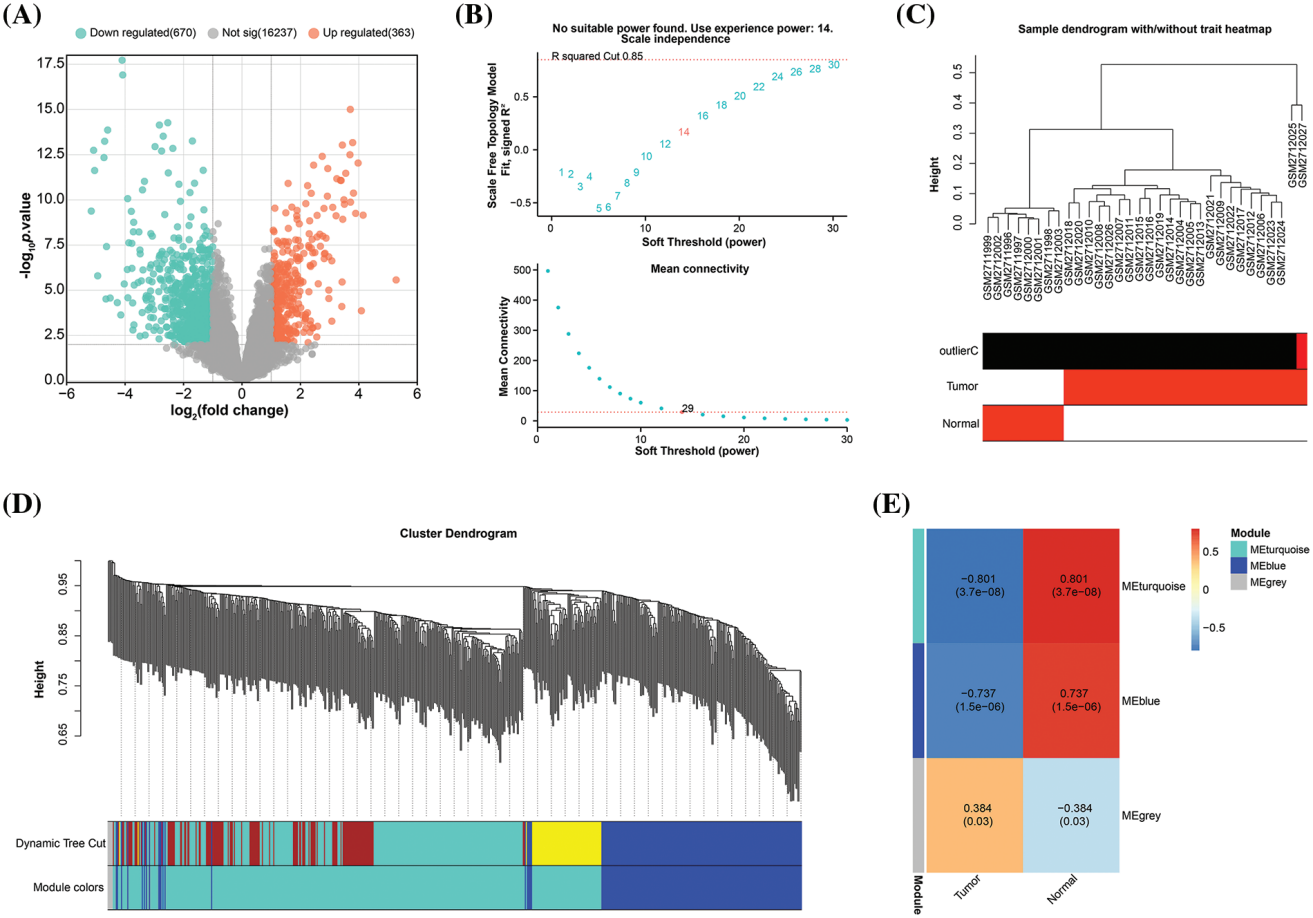
Construction of gene co-expression network. (A) Volcano plot, statistical significance of difference (negative log_10_-adjusted *p*-value; *y*-axis), and amount of change (log_2_ fold change; *x*-axis) for DEGs in the GSE101685 dataset. Each dot represents an individual gene. Orange dots indicate DEGs that are up-regulated, whereas green dots indicate down-regulated DEGs. (B) Graphical representation of the scale-free fit exponential analysis for various soft threshold powers (β). The scale-free fit index (*y*-axis) is shown against the soft-thresholding power (*x*-axis) in the above graph, which helps in choosing the optimal soft threshold. The mean connection (*y*-axis) for different soft-threshold powers (*x*-axis) is displayed in the picture below. (C) Trait heatmap of the dendrogram of 32 samples in the GSE101685 dataset. The heatmap provides a visual summary of the sample traits, with clustering patterns revealing relationships among samples. The vertical axis of the sample dendrogram is the standardised distance, which indicates the similarity between the different samples. (D) Cluster dendrogram of gene modules derived from hierarchical clustering. Each color represents a different module and the branches of the dendrogram group genes with similar expression profiles. The “Height” axis is a standardised unit of measurement in hierarchical cluster analysis, where height indicates the distance or similarity between data points. (E) Heatmap of correlations between gene modules and clinical features of HCC, with numbers in modules showing correlation coefficients and *p*-values.

A total of 1033 DEGs from the GSE101685 dataset using the GEO2R tool. Of these, 363 were up-regulated DEGs, and 670 were down-regulated DEGs (Fig. 1A). The probes were then placed into modules using the WGCNA package on R through average linkage clustering (Fig. 1B–E). A power of β = 14 (R^2^ = 0.85 without scale) was selected as a soft threshold, aiming to ensure a scale-free network, resulting in the identification of 2 modules (excluding the grey module). After comparing the correlation between the two modules and the two sets of samples in the GSE101685 dataset, it was discovered that the turquoise module (consisting of 452 genes) had the highest correlation with both sets of samples, with a correlation coefficient of 0.801.

### Prognostic analysis of 9 signature genes in the key module

**Figure 2:**
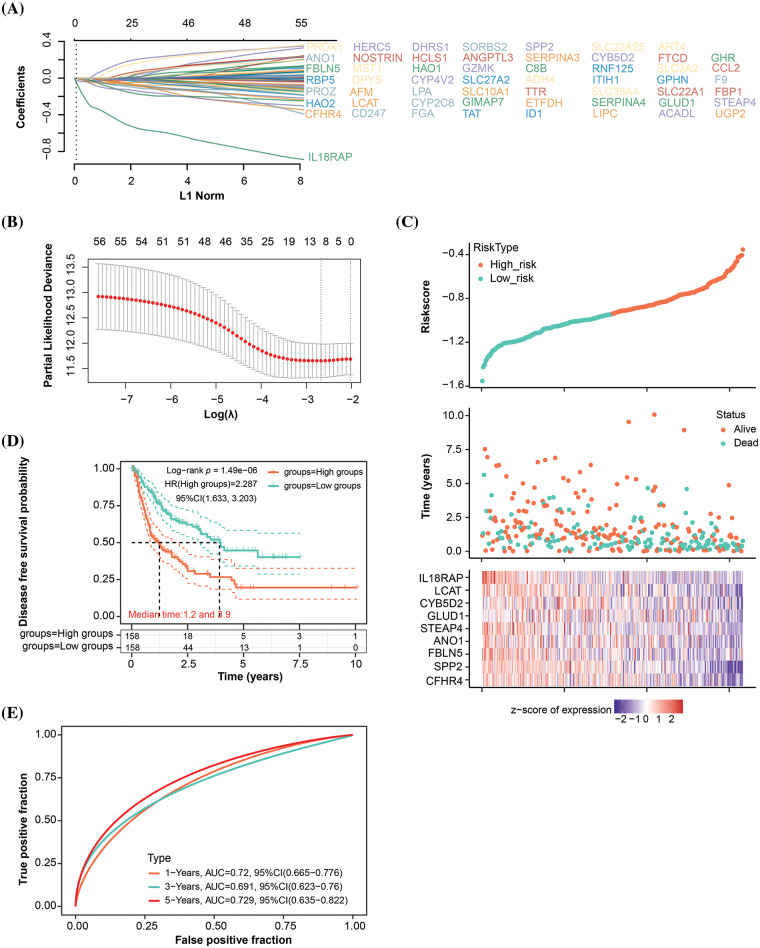
Signature prognostic model for the turquoise module. (A) Distribution of LASSO coefficients for genes with significant survival analysis results in the turquoise module. Each line corresponds to an individual gene, with the path of the coefficient profile depicted against the log (λ) sequence (The genes shown in (A) correspond to the genes in the provided Table S2.). (B) Partial likelihood deviation plot in a LASSO Cox regression model *vs*. log (λ). The plot is utilized to select the optimal parameter (λ), where the model achieves the smallest cross-validated error. The ideal values according to the 1-SE and minimum criterion are indicated by the two vertical lines. (C) The distribution of risk scores, survival time, and survival status are displayed in the risk score analysis for the chosen dataset samples. (D) KM survival curve analysis using the median risk score as a stratification tool to identify high- and low-risk groups. The log-rank test is used to determine the difference in survival probability between the two groups. (E) The 1-year, 3-year, and 5-year OS prediction accuracy is shown by the AUC values on the ROC curve of the landmark prognostic model.

After identifying the turquoise module as a key module, we conducted a DFS prognostic analysis on the genes within this module. As evident from the results in Table S2, a total of 56 genes showed significant prognosis. Subsequently, we performed LASSO Cox regression analysis on these 56 genes and identified 9 signature prognostic genes according to the optimal lambda value (lambda.min = 0.0688) (Fig. 2A,B) (The genes shown in (A) correspond to the genes in the provided Table S2.). The risk score for these genes was determined as follows: Riskscore = (−0.0212)*CFHR4 + (−0.0405)*SPP2 + (−0.0021)*FBLN5 + (−0.0157)*ANO1 + (−0.0261)*STEAP4 + (−0.0012)*GLUD1 + (−0.0336)*CYB5D2 + (−0.0321)*LCAT + (−0.2391)*IL18RAP. According to the risk model study, the high-risk group outperformed the low-risk group in terms of survival and death (Fig. 2C). KM survival analysis results, presented in Fig. 2D, elucidated that the median survival time for the high-risk group was notably shorter, at 1.2 years, compared to 3.9 years for the low-risk group, with an HR of 2.287 (>1), indicating a significant reduction in DFS probability for the high-risk group. The ROC curve analysis further accentuated the predictive accuracy of the risk model, especially pronounced at the 5-year mark (AUC = 0.729) (Fig. 2E). Our research concluded by highlighting the important prognostic potential of these nine genes.

### Nomogram analysis of key prognostic variables

**Figure 3:**
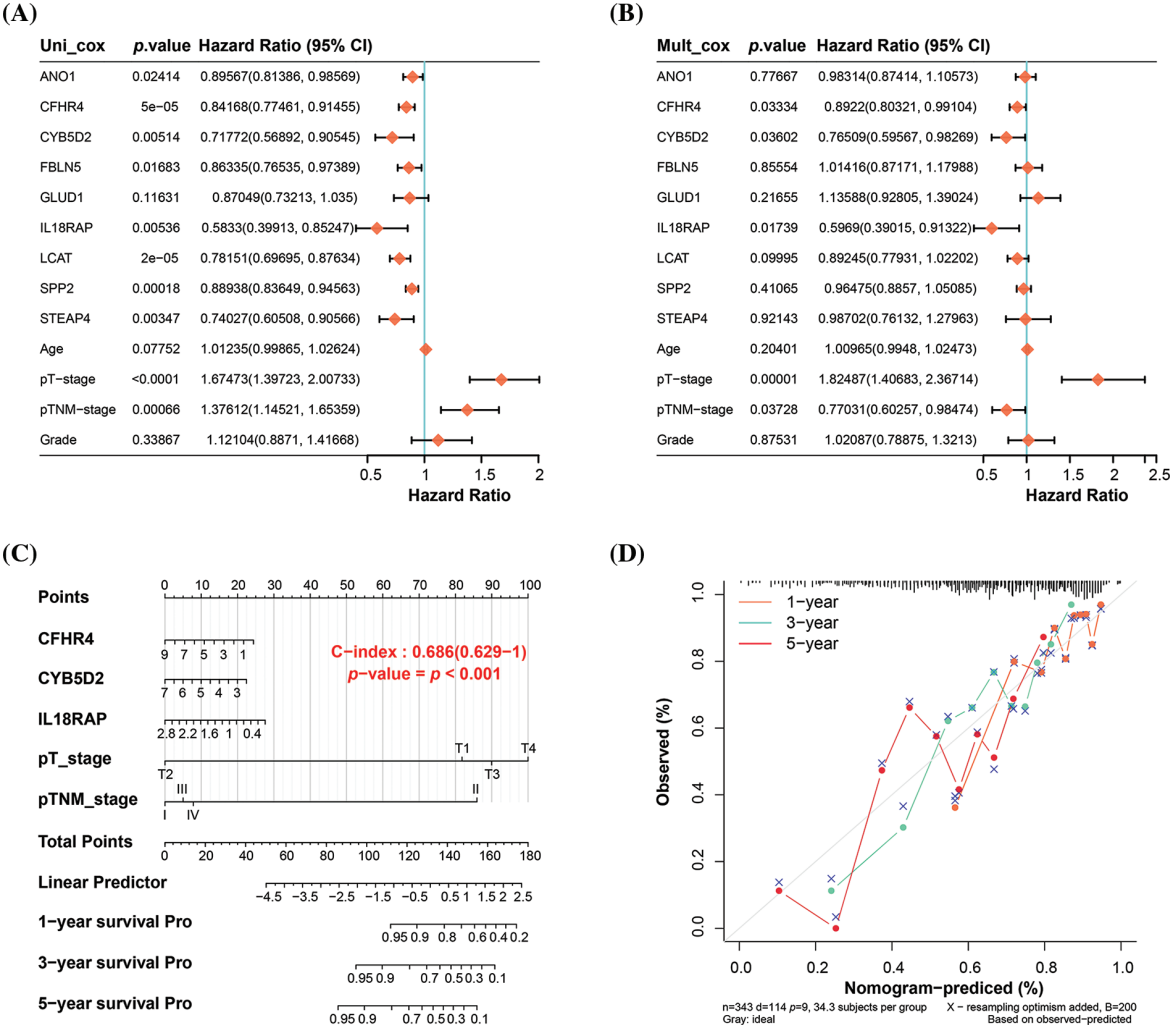
Nomogram analysis of key prognostic variables. (A) Univariate and (B) multivariate Cox regression analysis of nine genes and four clinical variables in the risk model yielded five significant variables: *CFHR4*, *CYB5D2*, *IL18RAP*, pT-stage, and pTNM-stage. (C) Nomogram analysis showing the significant predictive power of the identified variables on patient survival at 1, 3, and 5 years, with the length of the line segments reflecting the magnitude of the effect of the variables on patient survival. (D) Calibration curves that validate the predictive accuracy of the nomograms, with the middle dashed line being the ideal calibration curve.

In the analysis of nine genes and four clinical variables in the risk model, we identified five variables with statistical significance (*p* < 0.05), namely *CFHR4*, *CYB5D2*, *IL18RAP*, pT stage, and pTNM stage (Fig. 3A,B). The nomogram analysis results indicated that these variables had significant predictive power on the 1-, 3-, and 5-year survival rates of patients, which was also confirmed by the calibration curve results (Fig. 3C,D).

### Inverse association between CYB5D2 and TGF-β expression in LIHC

**Figure 4:**
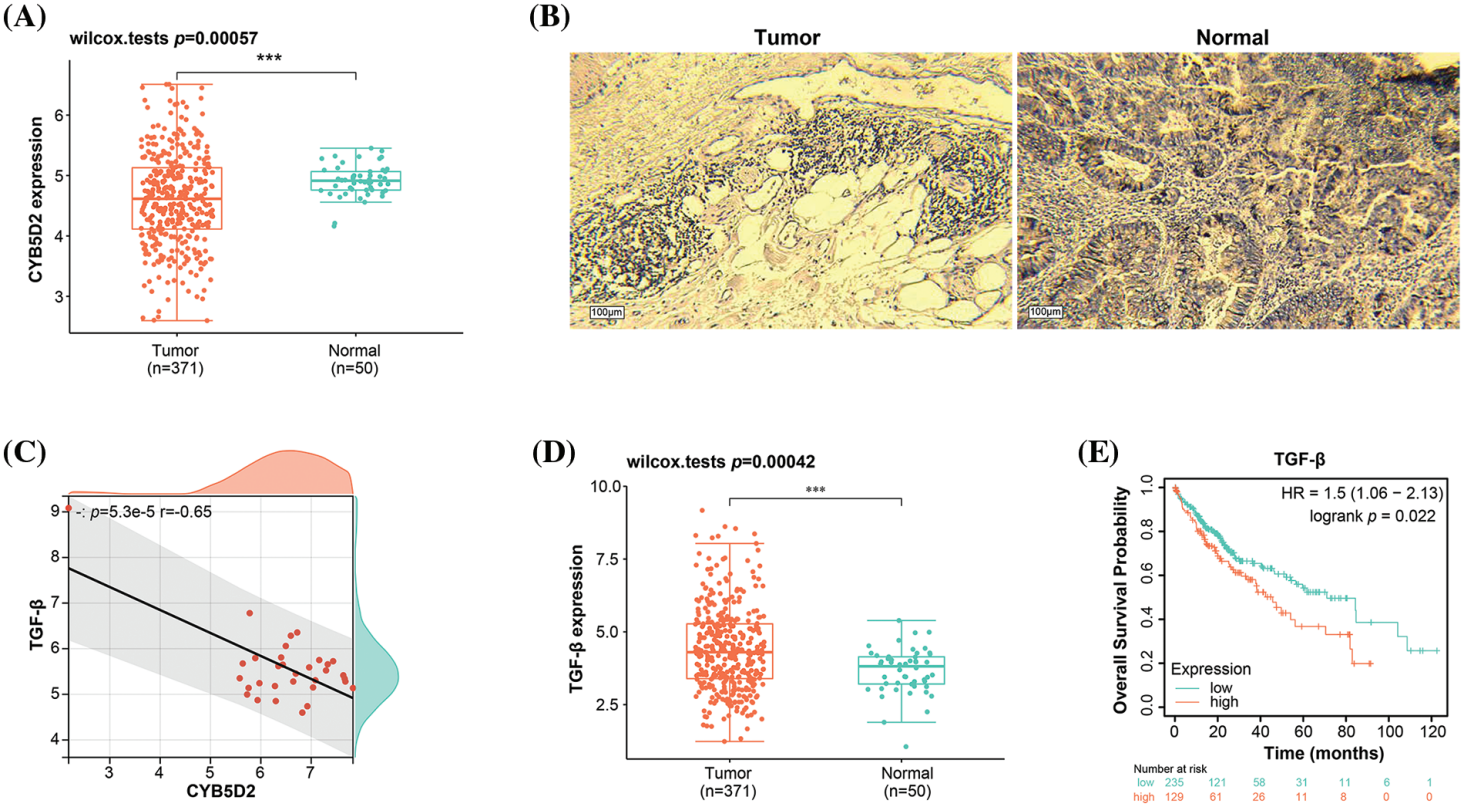
Expression levels and correlation analysis of *CYB5D2* and *TGF-β* in LIHC. (A) Boxplot, Wilcoxon detection of *CYB5D2* expression in TCGA-LIHC samples. ***p < 0.001. (B) Immunohistochemistry of *CYB5D2* in HCC tumor samples and normal samples. (C) Scatter plot, correlation analysis between *CYB5D2* and *TGF-β* expression levels, the correlation coefficient (r) is −0.65. (D) Boxplot, Wilcoxon detection of *TGF-β* expression in TCGA-LIHC samples. ***p < 0.001. (E) The correlation between *TGF-β* differential expression and LIHC OS prognosis is represented by the KM survival curve. OS probability is the vertical axis, while survival duration is the horizontal axis.

From the prognostic genes identified through nomogram analysis, *CYB5D2*, *CFHR4*, and *IL18RAP* have already been substantiated as participating in the pathogenesis of HCC. In this investigation, we chose *CYB5D2* as the hub gene to examine its relationship to HCC. Fig. 4A illustrated a noticeable underexpression of *CYB5D2* in LIHC, hinting towards a potential role in disease manifestation. These findings are further confirmed by immunohistochemistry results. Compared to normal liver tissue, the staining intensity of *CYB5D2* in tumor tissue was weakened, indicating a lower expression level in tumor tissue (Fig. 4B). Correlation analysis revealed a significant negative association (r = −0.65) between *CYB5D2* and *TGF-β* (Fig. 4C). To further substantiate our findings, we employed the Wilcoxon test, which confirmed *TGF-β* overexpression in LIHC (Fig. 4D,E). *TGF-β* overexpression was associated with a poorer OS prognosis, suggesting a potential prognostic role for *TGF-β* in LIHC.

### Downregulation of CYB5D2 and upregulation of TGF-β may be associated with malignant progression of HCC

**Figure 5:**
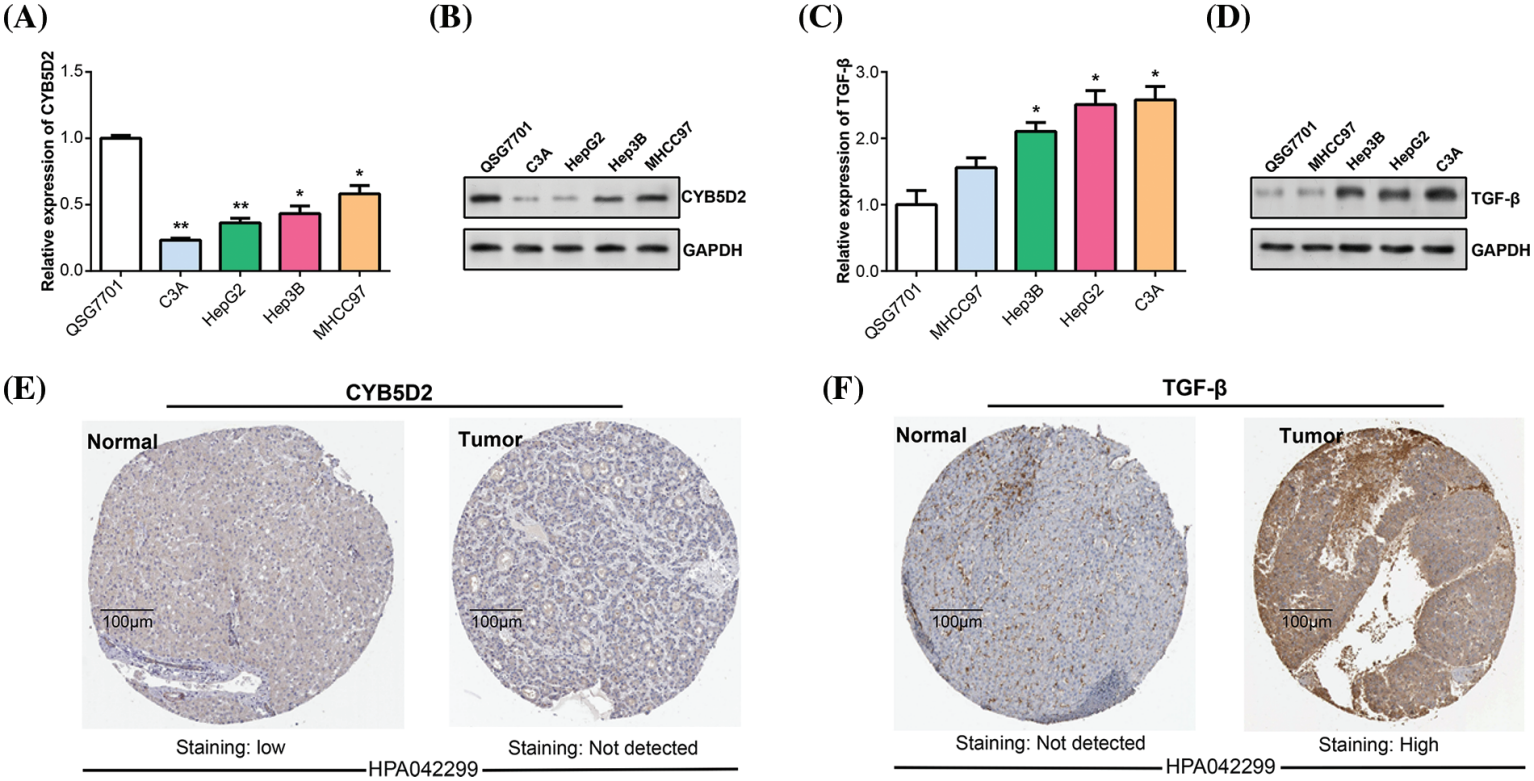
The expression of *CYB5D2* and *TGF-β* in HCC was detected *in vitro*. (A and B) qRT-PCR and WB were used to determine the *CYB5D2* expression levels in human normal liver cells (QSG7701) and HCC cell lines (C3A, HepG2, Hep3B, and MHCC97). (C and D) qRT-PCR and WB were used to determine the *TGF-β* expression levels in human normal liver cells (QSG7701) and HCC cell lines (C3A, HepG2, Hep3B, and MHCC97). (E and F) Immunohistochemical staining of *CYB5D2*/*TGF-β* expression in clinical samples of liver cancer. Images demonstrating the expression of *CYB5D2*/*TGF-β* in liver cancer tissues and nearby normal tissues are displayed. The brown color indicates positive staining. *p < 0.05, **p < 0.01.

By using the qRT-PCR and WB method *in vitro* experiments, we detected the *CYB5D2* and *TGF-β* expression levels in HCC cells. In C3A and HepG2, *CYB5D2* mRNA and protein levels were found to be much lower than in QSG7701 (Fig. 5A,B). In contrast, qRT-PCR and WB confirmed that C3A and HepG2 cells had elevated *TGF-β* expression levels (Fig. 5C,D). The HPA database also analyzed the levels of *CYB5D2* and *TGF-β* in HCC tissues. Among them, no staining signal was detected for *CYB5D2* in tumor tissue, and *TGF-β* showed high staining intensity in tumor tissue (Fig. 5E,F). This suggested that whilst *TGF-β* expression is markedly elevated in tumor tissues, *CYB5D2* expression is low in malignant tissues. Therefore, we speculated that the downregulation of *CYB5D2* and the upregulation of *TGF-β* may be related to the malignant progression of HCC.

### CYB5D2 overexpression inhibits HCC cell proliferation through the cell cycle

**Figure 6:**
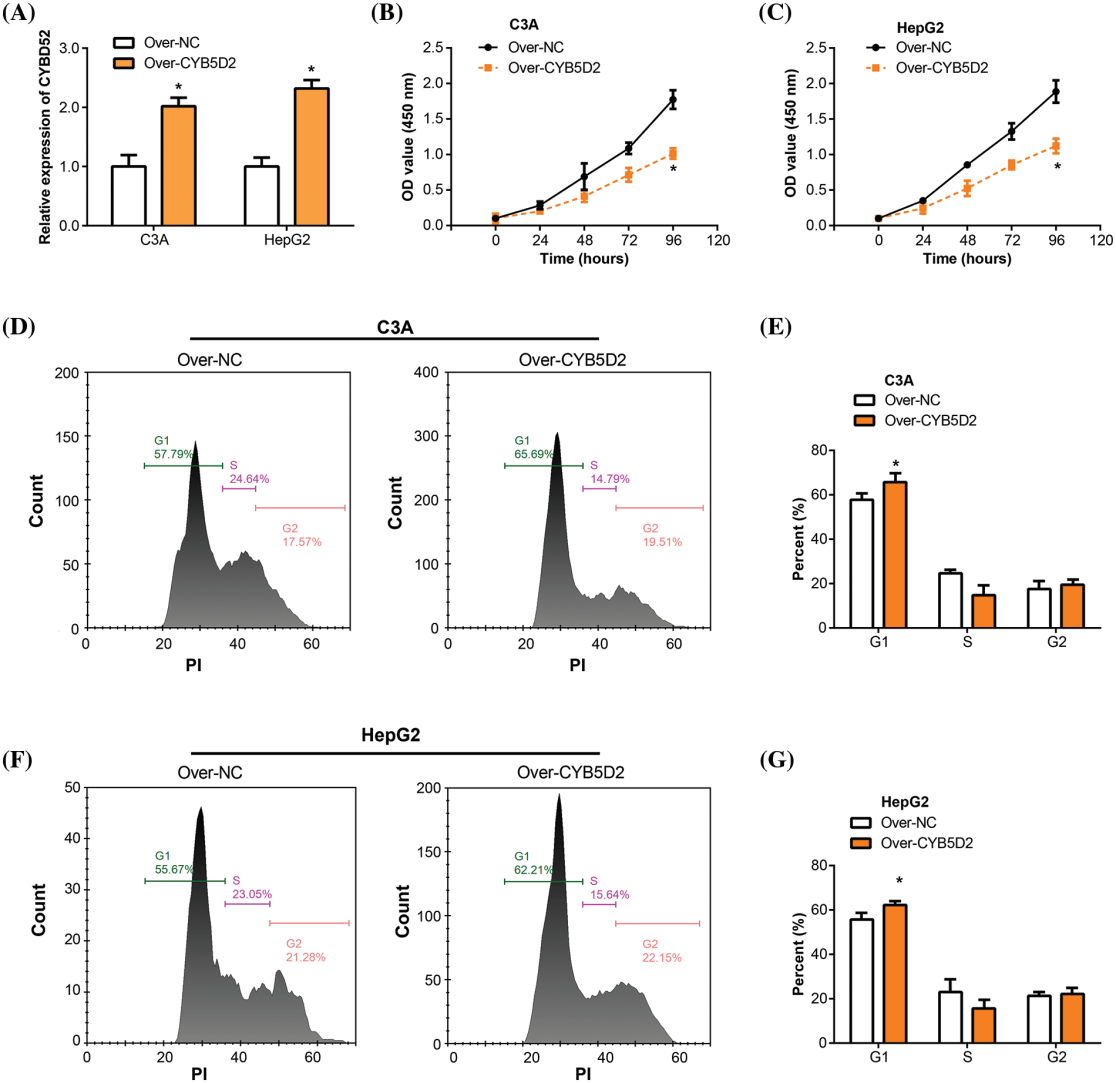
Overexpression of *CYB5D2* inhibits proliferation and induces cell cycle arrest in C3A and HepG2 cells. (A) *CYB5D2* mRNA expression in C3A and HepG2 cells following transfection with *CYB5D2* plasmid or empty vector control was examined using qRT-PCR. (B and C) The CCK-8 experiment demonstrates the growth of HepG2 and C3A cells following transfection with either the *CYB5D2* plasmid or an empty vector control. (D–G) Cell cycle distribution in C3A and HepG2 cells following transfection with either the *CYB5D2* plasmid or an empty vector control was examined using flow cytometry. *p < 0.05.

In our study, overexpression experiments were conducted by transfecting the *CYB5D2* plasmid into C3A and HepG2 cells. qRT-PCR analyses confirmed the upregulation of *CYB5D2* mRNA level post-transfection (Fig. 6A). Subsequently, the impact of *CYB5D2* overexpression on cell proliferation was assessed using the CCK-8 assay. Results demonstrated a significant reduction in the proliferation rates of both C3A and HepG2 cells following *CYB5D2* overexpression (Fig. 6B,C). Further analysis, as depicted in Fig. 6D–G, revealed that *CYB5D2* overexpression led to cell cycle arrest in the G1 phase, with an increase from 57.79% to 65.69% in C3A cells and from 55.67% to 62.21% in HepG2 cells. These findings collectively implied that *CYB5D2* overexpression inhibits cell proliferation and induces cell cycle arrest, providing insight into the potential therapeutic implications of targeting *CYB5D2* in HCC.

### Overexpression of CYB5D2 inhibits HCC cell migration and invasion

**Figure 7:**
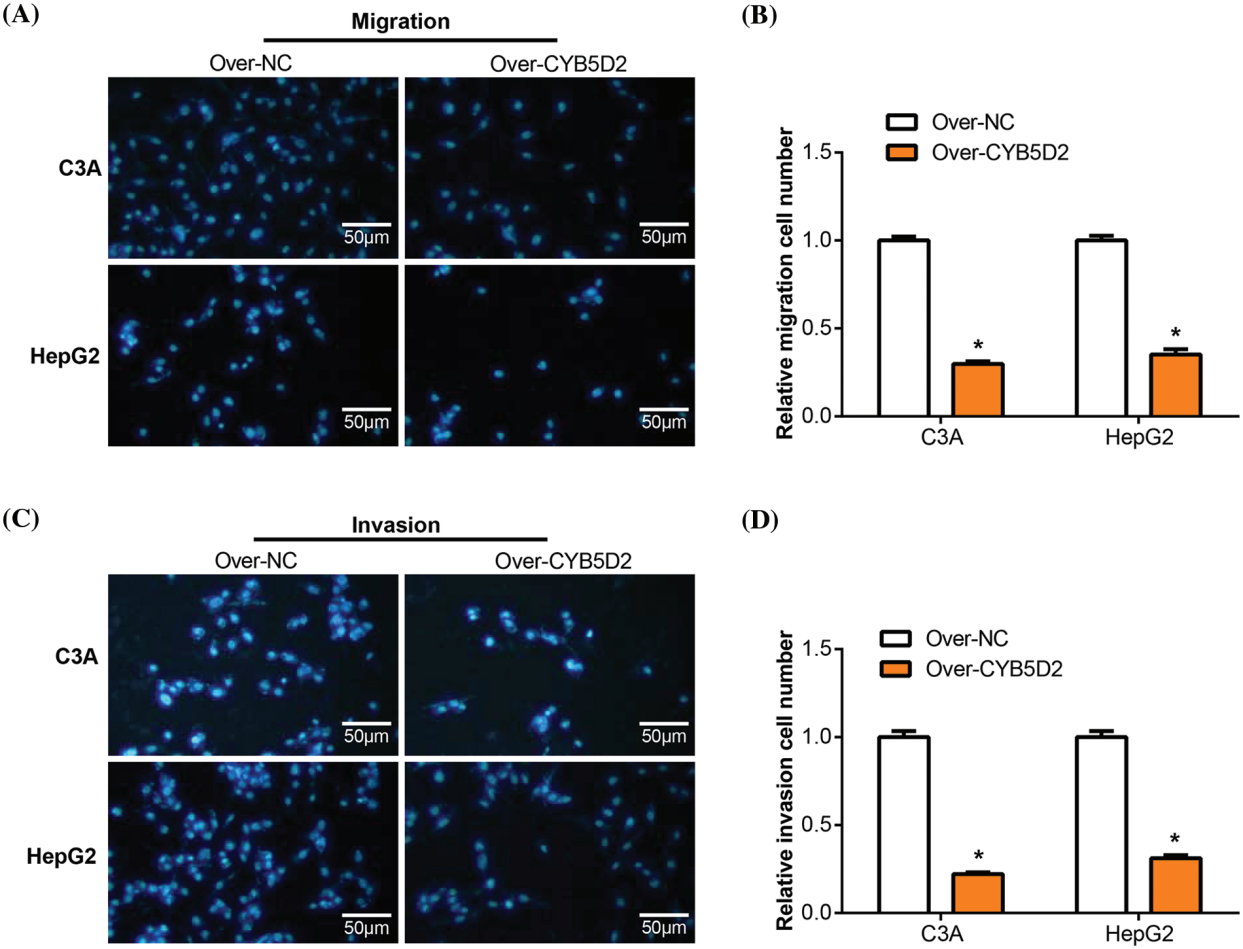
Overexpression of *CYB5D2* regulates the migration and invasion of C3A and HepG2 cells. (A–D) Transwell tests for C3A and HepG2 cells transfected with the *CYB5D2* plasmid or empty vector control to measure invasion and migration. The left panel displays representative microscopic pictures of the Transwell test findings, while the right panel displays bars that represent the quantification of migrating and invading cells. (A) and (B) were assayed without Matrigel; Figures C and D were assayed with Matrigel. Scale bars = 50 μm. *p < 0.05.

Transwell studies were then used to assay the effect of *CYB5D2* overexpression on the migration and invasion of C3A and HepG2 cells. The group with *CYB5D2* overexpression exhibited a significant decrease in the number of migrating and invading cells compared to the control group (Fig. 7A–D). It was demonstrated that overexpression of *CYB5D2* prevented the migration and invasion capabilities of C3A as well as HepG2 cells.

### TGF-β reverses the regulation of EMT markers and tumor progression by CYB5D2 overexpression in HCC

**Figure 8:**
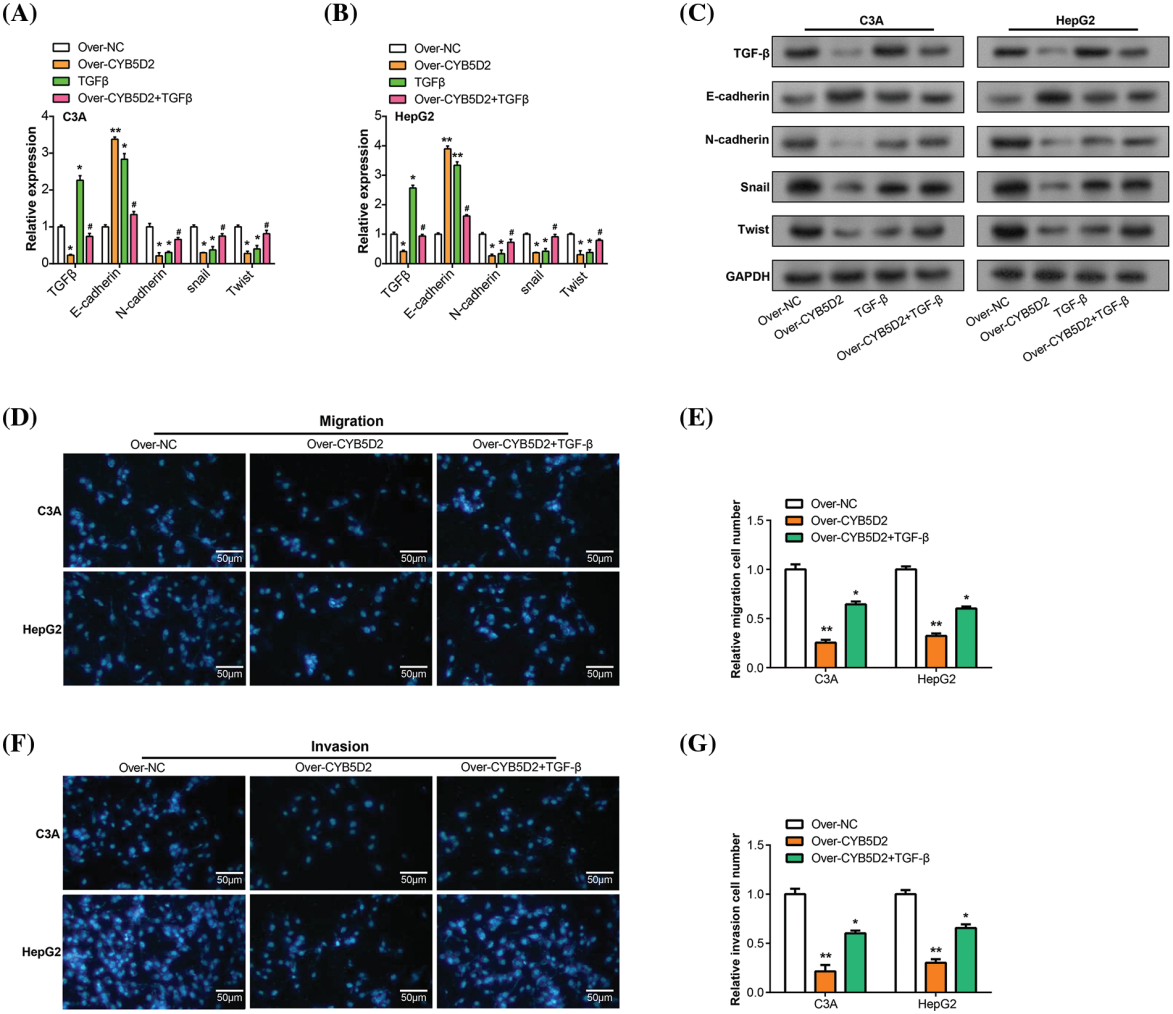
Regulation of *CYB5D2* and *TGF-β* on the expression of EMT-related factors and the migration, and invasion ability of C3A and HepG2 cells. (A and B) EMT-related variables were analyzed by qRT-PCR in three groups: control, over-*CYB5D2*, *TGF-β*, and over-*CYB5D2*+*TGF-β*. (C) WB analysis of EMT-related factors in control, over-*CYB5D2*, *TGF-β*, and over-*CYB5D2*+*TGF-β* groups. (D–G) Transwell detection of the effects of *TGF-β* and over-*CYB5D2* on C3A and HepG2 cell invasion and migration. The left panel displays representative microscopic pictures of the Transwell test findings, while the right panel displays bars that represent the quantification of migrating and invading cells. Scale bars = 50 μm. *p < 0.05, **p < 0.01.

The EMT plays a pivotal role in the development and metastasis of HCC [18,19], with key markers such as *E-cadherin*, and *N-cadherin* being closely linked to tumor progression [20]. To investigate the regulatory roles of *CYB5D2* and *TGF-β* on these EMT-associated factors, we segregated C3A and HepG2 cells into four groups: control (over-NC), *CYB5D2* overexpression (over-*CYB5D2*), *TGF-β*, and *CYB5D2* overexpression with *TGF-β* (over-*CYB5D2*+*TGF-β*). qRT-PCR analysis revealed a significant increase in E-cadherin expression in the over-*CYB5D2* group as compared to the control. However, with the addition of *TGF-β*, its expression level decreased. Conversely, the expression levels of N-cadherin, Snail, and Twist were downregulated by *CYB5D2* overexpression, with their levels rising following *TGF-β* treatment, as evidenced by qRT-PCR and corroborated by WB analysis (Fig. 8A–C). Based on these findings, we conducted Transwell rescue experiments and discovered that overexpression of *CYB5D2* significantly reduced the migratory and invasive behavior of C3A and HepG2 cells compared to the control group. The addition of *TGF-β* partially counteracted this inhibitory effect (Fig. 8D–G). Thus, our results suggested that *CYB5D2* inhibited both the migration and invasion of HCC cells and modified the expression of EMT-related proteins. *TGF-β* might play a role in reversing the inhibitory effects of *CYB5D2* on C3A and HepG2.

### Overexpression of CYB5D2 exhibits anticancer activity in vivo

**Figure 9:**
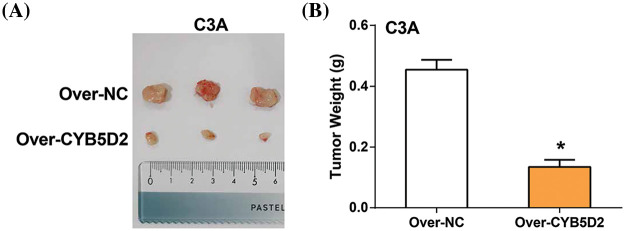
Impact of *CYB5D2* overexpression on tumor growth in C3A cell xenografts. (A) Measurement of tumor size changes in nude mice transfected with C3A cells after *CYB5D2* overexpression. (B) Observation of changes in tumor weight with or without overexpression of *CYB5D2*. *p < 0.05.

The study evaluated the *in vivo* effects of overexpressing *CYB5D2* were assessed using a xenograft model of HCC in nude mice. The results showed a significant reduction in tumor size and weight after *CYB5D2* overexpression in the nude mouse model (Fig. 9A,B). These findings underscored the impact of *CYB5D2* modulation on tumorigenesis in HCC.

## Discussion

In cases of HCC, patients are often diagnosed at an advanced stage, which precludes surgical resection. Therefore, the search for reliable biomarkers has become critical to assist in the early diagnosis of hepatocellular carcinoma and accurate prognosis prediction for patient survival [21]. The onset of liver cancer is a complex multi-factor process [22]. Excessive drinking, viral infections (hepatitis B or C), and worsening of liver cirrhosis can all lead to HCC [23,24]. Under normal circumstances, the ratio of HCC in men to women is 7–10:1. This means that the number of male HCC patients is 7–10 times higher than that of females. More than 90% of HCC cases are related to hepatic inflammation and damage. Chronic unresolved inflammation is linked to persistent liver injury and concurrent regeneration, which can lead to in fibrosis, cirrhosis, and eventually HCC [25,26]. Currently, HCC can be diagnosed through non-invasive imaging techniques such as magnetic resonance imaging and computed tomography [27,28].

Currently, the use of WGCNA and LASSO regression algorithms for prognostic and target analysis of HCC has become increasingly common. Yang et al. performed bioinformatics analysis of HCC-related data in TCGA and GEO based on the above two datasets, and found 4 macrophage-related genes (*CDCA8*, *CBX2*, *UCK2*, and *SOCS2*) with good prognostic independence in HCC, which are expected to be the potential prognostic target [29]. Shan et al. conducted a WGCNA analysis on the DEGs in the GSE22058 and GSE54238 datasets, identified key modules, and developed a prognostic model using the LASSO regression algorithm. Through this meticulous analysis, UBA1 was identified as a unique biomarker for liver cancer diagnosis and prognosis [30]. Ding et al. used the WGCNA and the LASSO algorithms to find that *MMP9* is highly correlated with the prognosis of HCC patients. They also found that the dual immunological signal of *MMP9* and CD8^+^ T cells can increase the survival rate of patients with HCC [31]. In summary, the research on the main goals and processes of HCC using the WGCNA and LASSO algorithms is convincing.

In our research, we constructed a gene co-expression network for DEGs in the GSE101685 dataset using WGCNA. We identified the turquoise module as a key gene module. Subsequently, survival analysis and LASSO Cox prognostic model and nomogram analysis were performed on the genes in these two modules, and three significant prognostic genes were identified, namely *CFHR4*, *CYB5D2*, and *IL18RAP*. This analysis highlights their potential role in HCC progression and patient prognostic outcomes. Research by Ding et al. pointed out that *CFHR4* is significantly low-expressed in HCC, which can lead to poor patient prognosis, and its level of expression and the extent of immune cell infiltration are also correlated [32]. Yu et al. further demonstrated the significant predictive value of *CFHR4* in HCC by showing a strong correlation between the expression of this gene and several clinicopathological factors that are strongly correlated with immune cell infiltration [33]. *CFHR4* has also been identified as one of the hepatocyte subtypes in extrahepatic metastasis of advanced HCC and may serve as a predictive target for resistance to the combination therapy of lenvatinib, *FOLFOX*, and toripalimab [34]. Li et al. identified five genes, including *IL18RAP*, that are associated with the prognosis of HCC. They also verified that there is a relationship between immune cell infiltration and immunotherapy sensitivity and the expression of these genes [35]. Ren et al. identified *CYB5D2* as a key prognostic gene for HCC through LASSO and multivariable Cox regression analysis on HCC-related data sets in public databases [36]. However, the precise mechanism of action of *CYB5D2* in HCC is still unknown and requires further research.

Our investigation revealed that *CYB5D2* is underexpressed in LIHC and exhibits a negative correlation with *TGF-β*, which is found to be highly expressed in LIHC and associated with adverse prognoses in HCC patients. Research has indicated that *TGF-β* is one of the main cytokines known to cause EMT and that it plays a significant role in the process [37]. *TGF-β* is an important inducer of EMT and plays a role in development, wound healing, and diseases such as fibrosis and cancer. During these processes, EMT is intertwined with changes in cell proliferation, differentiation, communication, and extracellular matrix remodeling, and is coordinately regulated by multiple signaling inputs, including *TGF-β* [38]. For instance, gangliosides catalyzed by ST3 beta-galactoside alpha-2,3-sialyltransferase 5 (ST3GAL5)-catalyzed gangliosides inhibit *TGF-β*-induced EMT via degradation of the *TGF-β* type I receptor (TβRI) [39]. The interaction and methylation of SMAD4 by the oncogene protein arginine methyltransferase 5 (PRMT5) are essential for driving *TGF-β1*-induced EMT and promoting metastasis in colorectal cancer (CRC) [40]. *TGF-β* can contribute to tumor growth, metastasis, and resistance to clinical therapy by activating certain transcription factors that downregulate epithelial indicators and upregulate mesenchymal markers [41]. A study discovered that *STAT3* induces EMT and liver cancer metastasis by positively regulating *TGF-β1*. Through the PI3K/Akt/Rac1 pathway, *MMP-8* and *TGF-β1* mutual activation induces EMT, which in turn promotes the development of HCC [42]. In addition, another study also pointed out that the *FCN2*/*TGF-β*/EMT axis is an important mechanism affecting HCC metastasis and affects the metastasis of HCC [43]. This study aimed to investigate the precise mechanism of action of *TGF-β* and *CYB5D2* in HCC using *in vitro* experiments. The results demonstrate that *CYB5D2* is downregulated in HCC cells, and overexpression of *CYB5D2* inhibits HCC cell growth and induces G1 arrest. *CYB5D2* and *TGF-β* jointly regulate the expression of EMT-related factors and the progression of HCC. Specifically, *CYB5D2* overexpression leads to upregulation of *E-cadherin* and downregulation of *N-cadherin*, *Snail*, and *Twist*, indicating that *CYB5D2* may function as an EMT inhibitor. This modulation of EMT-related factors corresponds to significant inhibition of HCC migration and invasion capabilities (the reduction in invasions may be attributed to factors such as reduced migration), supporting the theory that *CYB5D2* could have tumor-suppressive effects in HCC. These effects were partially reversed upon the addition of *TGF-β*, suggesting a potential interaction between *TGF-β* and *CYB5D2*. In addition, tumor xenograft experiments also confirmed that *CYB5D2* overexpression significantly inhibited tumor growth in mice. Overall, *CYB5D2* plays an important role in the formation and progression of HCC and may have a significant impact on preventing EMT and tumor growth of HCC by regulating key EMT indicators and counteracting the tumorigenic effects of *TGF-β*.

Although our study preliminarily explored relevant mechanisms at the molecular level, it is important to recognize the limitations that exist. Our study had not yet addressed how to effectively regulate the development and progression of HCC. The molecular-level data we have obtained have only a basic level of evidence and are not sufficient to construct a strong argument. However, this study highlighted the potential of *CYB5D2*-targeted therapies as innovative treatments for HCC and provided important clues for developing new therapeutic directions.

## Conclusion

In summary, this study utilized bioinformatics methods to analyze DEGs in GSE101685 and identified *CYB5D2* as the hub gene associated with HCC prognosis. It was found that *CYB5D2* is significantly low-expressed in HCC and negatively correlated with *TGF-β*. *TGF-β* is a key factor that promotes tumor progression and metastasis through EMT. *CYB5D2* overexpression can partially counteract the inhibition of HCC cell proliferation, migration, and invasion as well as the regulation of EMT markers by *TGF-β*, suggesting a complex interaction between *CYB5D2* and *TGF-β* in regulating HCC progression. *In vivo* experiments further confirmed that *CYB5D2* overexpression can significantly reduce tumor growth, indicating its potential application value as a therapeutic target for HCC.

## Supplementary Materials





## Data Availability

The datasets used and/or analyzed during the current study are available from the corresponding author upon reasonable request.

## Supplementary Materials

The supplementary material is available online at https://doi.org/10.32604/or.2024.050125.
