# Supplementary material for: CYB5D2 inhibits the malignant progression of hepatocellular carcinoma by inhibiting TGF-β expression and epithelial-mesenchymal transition
Source: Oncol Res. 2025 Feb 28;33(3):709–22. doi: 10.32604/or.2024.050125 (PMC11915040; doi:10.32604/or.2024.050125)
Supplement: Supplementary file 1 [file OncolRes-33-50125-s001.docx]

**Supplementary Table S1.** **Primer sequences for qRT-PCR.**

| **Gene** | **Forward primers (5′-3′)** | **Reverse primers (5′-3′)** |
| --- | --- | --- |
| *CYB5D2* | GACCGGGGACTGTTCTGAAG | TAGAACCGTCCTGTCACCCT |
| *TGF-β* | ATACGCCTGAGTGGCTGTCT | TCTCTGTGGAGCTGAAGCAA |
| *E-cadherin* | CTGAGAACGAGGCTAACG | GTCCACCATCATCATTCAATAT |
| *N-cadherin* | CACTGCTCAGGACCCAGAT | TAAGCCGAGTGATGGTCC |
| *Snail* | GGCTCCTTCGTCCTTCTCCTCTAC | CCAGGCTGAGGTATTCCTTGTTGC |
| *Twist* | GTCCGCAGTCTTACGAGGAG | GCTTGAGGGTCTGAATCTTGCT |
| *GAPDH* | GAAGGTGAAGGTCGGAGTC | GAAGATGGTGATGGGATTTC |
