# Supplementary material for: CYB5D2 inhibits the malignant progression of hepatocellular carcinoma by inhibiting TGF-β expression and epithelial-mesenchymal transition
Source: Oncol Res. 2025 Feb 28;33(3):709–22. doi: 10.32604/or.2024.050125 (PMC11915040; doi:10.32604/or.2024.050125)
Supplement: Supplementary file 2 [file OncolRes-33-50125-s002.docx]

**Supplementary Table S2.** **Batch survival analysis of genes in the turquoise module.**

| **Genes** | ***p*.value** | **HR** | **Low 95%CI** | **High 95%CI** |
| --- | --- | --- | --- | --- |
| SLC22A1 | 0.000619207 | 0.559505149 | 0.401238463 | 0.780199411 |
| HAO2 | 0.001995568 | 0.592933682 | 0.425664177 | 0.825933611 |
| CFHR4 | 0.002145663 | 0.595455338 | 0.427635959 | 0.829132941 |
| GHR | 0.042691023 | 0.711526777 | 0.511977692 | 0.98885237 |
| SPP2 | 0.001356366 | 0.581944769 | 0.417873041 | 0.810436857 |
| F9 | 0.013788709 | 0.660709616 | 0.47507869 | 0.918873453 |
| SLC38A4 | 0.025149495 | 0.686346459 | 0.49370091 | 0.954163649 |
| TTR | 0.02477228 | 0.685185446 | 0.492559529 | 0.953141839 |
| RNF125 | 0.02589405 | 0.687007165 | 0.493768734 | 0.955870254 |
| SLC10A1 | 0.007451545 | 0.637349516 | 0.458244474 | 0.886457836 |
| C8B | 0.014255191 | 0.662872437 | 0.47711306 | 0.92095544 |
| ACADL | 0.020658594 | 0.677254569 | 0.486865177 | 0.942096032 |
| LPA | 0.001113104 | 0.575086369 | 0.412374933 | 0.801999115 |
| ADH4 | 0.00130609 | 0.578676649 | 0.414565573 | 0.807753191 |
| FBP1 | 0.00221047 | 0.595844748 | 0.427682873 | 0.830126682 |
| ID1 | 0.026702401 | 0.687534305 | 0.493602526 | 0.957660051 |
| AFM | 0.027127213 | 0.689621131 | 0.495975132 | 0.958873286 |
| SERPINA4 | 0.005957143 | 0.628789885 | 0.451756529 | 0.875198684 |
| DHRS1 | 0.010455034 | 0.649569093 | 0.466865678 | 0.90377174 |
| PROZ | 0.04496469 | 0.713045182 | 0.512309326 | 0.992434464 |
| ART4 | 0.025535279 | 0.686106216 | 0.49294786 | 0.954952395 |
| CCL2 | 0.044102658 | 0.712384374 | 0.5120559 | 0.991086122 |
| TAT | 0.026753013 | 0.688933026 | 0.495442225 | 0.957990035 |
| UGP2 | 0.00154892 | 0.585178368 | 0.419947565 | 0.815420187 |
| FBLN5 | 0.021591604 | 0.679340837 | 0.488475427 | 0.94478442 |
| GZMK | 0.04459807 | 0.712810623 | 0.512266488 | 0.991864577 |
| ANO1 | 0.032235395 | 0.697979845 | 0.502252269 | 0.969982405 |
| SLC2A2 | 0.001862621 | 0.591165461 | 0.424519245 | 0.823229115 |
| HCLS1 | 0.006273326 | 0.629085146 | 0.451192549 | 0.877115817 |
| SLC27A2 | 0.029190826 | 0.692832995 | 0.49819829 | 0.963507039 |
| LIPC | 0.021373305 | 0.677506204 | 0.48630367 | 0.943884828 |
| HAO1 | 0.039773063 | 0.707280737 | 0.508412341 | 0.983937643 |
| STEAP4 | 0.024586416 | 0.684699414 | 0.492115514 | 0.952648869 |
| FGA | 0.024121402 | 0.683638602 | 0.491219891 | 0.951430809 |
| DPYS | 0.025356202 | 0.686808491 | 0.494094201 | 0.954688201 |
| ANGPTL3 | 0.049049824 | 0.717437748 | 0.515429038 | 0.998618401 |
| RBP5 | 0.029401937 | 0.691476436 | 0.496132914 | 0.963733001 |
| ETFDH | 0.024031686 | 0.683533831 | 0.491182266 | 0.951212067 |
| GLUD1 | 0.00434438 | 0.615547464 | 0.440998814 | 0.859182992 |
| HERC5 | 0.028806243 | 1.449235921 | 1.039131492 | 2.021192477 |
| CYP2C8 | 0.038609102 | 0.706776095 | 0.508698207 | 0.981981931 |
| SLC22A25 | 0.034087057 | 0.699934097 | 0.503206059 | 0.973572818 |
| NOSTRIN | 0.045206231 | 0.713233987 | 0.51238706 | 0.99280946 |
| GPHN | 0.024456068 | 0.684646514 | 0.49219233 | 0.952353015 |
| SERPINA3 | 0.015148225 | 0.663926999 | 0.47706555 | 0.92398007 |
| GIMAP7 | 0.010104805 | 0.648500668 | 0.466220362 | 0.902047939 |
| CYB5D2 | 0.001988843 | 0.591796852 | 0.424377112 | 0.82526485 |
| SORBS2 | 0.047940529 | 0.716990984 | 0.515628928 | 0.99698842 |
| PROX1 | 0.021544793 | 0.678072458 | 0.486844441 | 0.944413083 |
| FTCD | 0.025758957 | 0.686873588 | 0.493736757 | 0.955560468 |
| ITIH1 | 0.000959566 | 0.570479189 | 0.40884311 | 0.796018074 |
| LCAT | 0.046728842 | 0.715507293 | 0.514437338 | 0.995166268 |
| IL18RAP | 0.008047814 | 0.63864987 | 0.458391508 | 0.889793222 |
| CYP4V2 | 0.03252772 | 0.698164687 | 0.502227724 | 0.970543653 |
| CD247 | 0.033804456 | 0.699402328 | 0.502733223 | 0.973008334 |
| MST1 | 0.042050711 | 0.709816089 | 0.510082667 | 0.987759265 |
